# Visualization of junctional epithelial cell replacement by oral gingival epithelial cells over a life time and after gingivectomy

**DOI:** 10.1038/s41598-019-44065-x

**Published:** 2019-05-21

**Authors:** Mayu Kato, Junichi Tanaka, Ryo Aizawa, Sara Yajima-Himuro, Tatsuaki Seki, Keisuke Tanaka, Atsushi Yamada, Miho Ogawa, Ryutaro Kamijo, Takashi Tsuji, Kenji Mishima, Matsuo Yamamoto

**Affiliations:** 1 Department of Periodontology, School of Dentistry, Showa University, 2-1-1 Kitasenzoku, Ohta-ku, Tokyo, 145-8515 Japan; 2 Division of Pathology, Department of Oral Diagnostic Sciences, School of Dentistry, Showa University, 1-5-8 Hatanodai, Shinagawa-ku, Tokyo, 142-8555 Japan; 3 Department of Biochemistry, School of Dentistry, Showa University, 1-5-8 Hatanodai, Shinagawa-ku, Tokyo, 142-8555 Japan; 4 Laboratory for Organ Regeneration, RIKEN Center for Biosystems Dynamics Research (BDR), Kobe, Hyogo, 650-0047 Japan; 5 Organ Technologies Inc., Tokyo, 101-0048 Japan

**Keywords:** Cellular imaging, Animal biotechnology, Cellular imaging, Animal biotechnology

## Abstract

Junctional epithelium (JE), which is derived from odontogenic epithelial cells immediately after eruption, is believed to be gradually replaced by oral gingival epithelium (OGE) over a lifetime. However, the detailed process of replacement remains unclear. The aim of the present study was to clarify the process of JE replacement by OGE cells using a green fluorescent protein (GFP)–positive tooth germ transplantation method. GFP-positive JE was partly replaced by OGE cells and completely replaced on day 200 after transplantation, whereas there was no difference in the expression of integrin β4 (Itgb4) and laminin 5 (Lama5) between JE before and after replacement by OGE cells. Next, GFP-positive JE was partially resected. On day 14 after resection, the regenerated JE consisted of GFP-negative cells and also expressed both Itgb4 and Lama5. In addition, the gene expression profile of JE derived from odontogenic epithelium before gingivectomy was partly different from that of JE derived from OGE after gingivectomy. These results suggest that JE derived from the odontogenic epithelium is gradually replaced by OGE cells over time and JE derived from the odontogenic epithelium might have specific characteristics different to those of JE derived from OGE.

## Introduction

Periodontitis is an inflammatory condition affecting the periodontal tissue, including the gingival epithelium, and has been reported to be associated with systemic diseases, including cardiovascular disease and diabetes^1,2^. The gingival epithelium consists of oral gingival epithelium (OGE), oral sulcular epithelium, and junctional epithelium (JE)^3–7^. The contribution of JE to infection prevention is distinctive, owing to its location on “the front lines” in the oral cavity, where it directly contacts the tooth surface by means of a complex hemidesmosome network^8–10^. The JE is designed to provide a seal around the teeth to defend the internal environment against bacterial infection from dental plaques and periodontal disease^6,8,11^. Turnover here is just 4–6 days; this is extremely fast, especially compared with the 6–12 days seen in the oral epithelium^12–14^. In addition, gingival crevicular fluid (GCF) is exuded by the periodontal tissue into the gingival sulci and pockets, where it mixes with saliva. GCF also contains molecules released by junctional epithelial cells, recruited neutrophils, and other immunocytes, including secretory components, cytokines, enzymes, lysozymes, lactoferrins, and complements, which provide anti-microbial action^15–19^. The JE has unique features, different from those of OGE cells.

In the developing tooth, two layers of cells, the inner layer of ameloblast and outer layer of cuboidal cells, remain as the reduced enamel epithelium covering the enamel surface after enamel formation is completed. The reduced enamel epithelium fuses with the OGE during tooth eruption into the oral cavity and is converted into JE^6,20,21^. Therefore, the JE immediately after eruption is thought to be derived from the odontogenic epithelium rather than the OGE. Importantly, we have revealed, using a bioengineered tooth system^22^, that the JE immediately after eruption is derived from the odontogenic epithelium^23^. The JE is believed to be gradually replaced by OGE cells over a lifetime^20,24–27^. However, to date, there have been no investigation to definitively clarify this replacement. In addition, another important issue is whether odontogenic epithelium–derived JE contributes to the regeneration of the JE after gingivectomy.

Here, using a tooth germ transplantation technique, we examined the possibility that JE derived from odontogenic epithelium is replaced by OGE cells after tooth eruption or after a partial gingivectomy. We demonstrated that the JE derived from odontogenic epithelium was replaced by OGE cells by time course study and the regenerated JE after gingivectomy originated from OGE cells. Further, the gene expression profiles between JE derived from odontogenic epithelium and OGE were compared.

## Results

### JE derived from odontogenic epithelium was replaced by OGE cells

**Figure 1:**
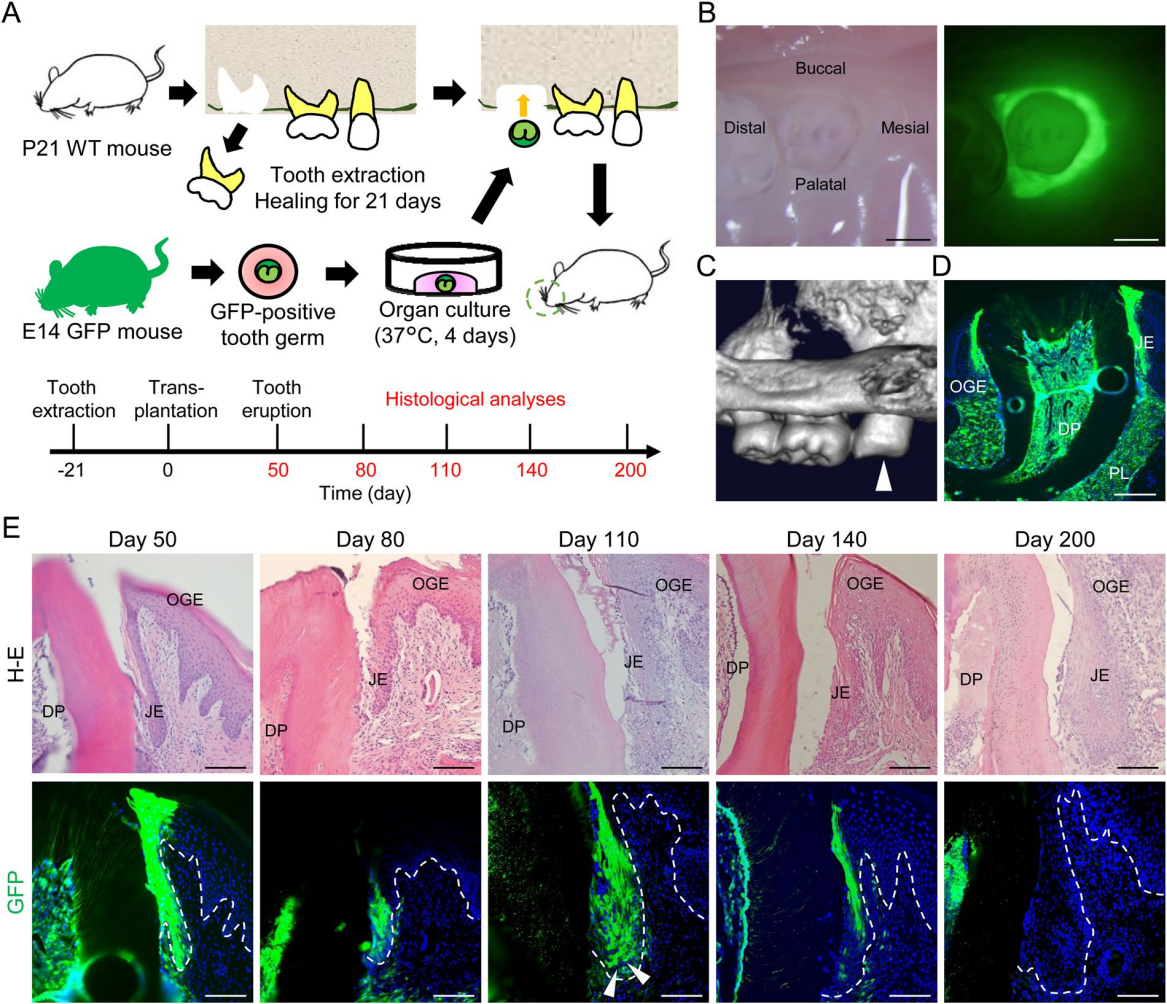
GFP-positive odontogenic cells sequentially decreased in JE. (**A**) Schema of the GFP-positive tooth germ transplantation method (top). Histological analysis of periodontal tissue around the erupted GFP-positive transplanted teeth in WT mice on day 50, 80, 110, 140, and 200 after transplantation (bottom). (**B**) Representative bright- (left) and dark-field (right) imaging of an erupted GFP-positive tooth in a WT mouse on day 50 after transplantation. Scale bars represent 500 μm. (**C**) Representative micro-3DCT image of the erupted GFP-positive tooth on day 50 after transplantation (white arrowhead). (**D**) Representative fluorescence image of the transverse section of periodontal tissue around the erupted GFP-positive tooth in the WT mouse on day 50 after transplantation. Scale bar represents 200 μm. Abbreviations: OGE, oral gingival epithelium; JE, junctional epithelium; DP, dental pulp; PL, periodontal ligament. (**E**) Hematoxylin and eosin (H&E) stained (upper row) and fluorescence images (lower row) of transverse sections of periodontal tissue around the erupted GFP-positive teeth in the WT mice on day 50, 80, 110, 140, and 200 after transplantation. From day 110 to 140, GFP-positive cells gradually decreased. On day 200, almost all JE cells were GFP-negative. Dotted lines show the basement membrane. Representative images were taken from three independent mouse samples (*n* = 3). Scale bars represent 100 μm. Abbreviations: OGE, oral gingival epithelium; JE, junctional epithelium; DP, dental pulp.

To clarify whether JE derived from odontogenic epithelium is replaced by OGE cells, green fluorescent protein (GFP)-positive tooth germs obtained from C57BL/6-Tg (CAG-EGFP) mice on embryonic day 14 (E14) were transplanted into the bone holes left after the extraction of the upper first molars of C57BL/6 N (wild type: WT) mice on postnatal day 21 (P21) (Fig. 1A). On day 50 after transplantation, i.e., after eruption, the JE around the transplanted tooth expressed GFP (Fig. 1B,C). Fluorescence analysis of frozen sections on day 50 showed that not only the JE but also the dental pulp and the periodontal ligament expressed GFP (Fig. 1D). Most cells of the JE, including the internal and external basal lamina (IBL and EBL, respectively) were found to be GFP-positive odontogenic cells on days 50 and 80 (Fig. 1E). On the other hand, on day 110, the number of GFP-positive cells decreased and was partly replaced by GFP-negative cells localized in the basal and suprabasal layers (white arrowheads). On day 140, GFP-positive cells were observed only in JE cells attached to the enamel surface. On day 200, no GFP-positive cells could be detected in the JE. However, GFP-positive cells were detected in the dental pulp at each time point (Fig. 1E).

**Figure 2:**
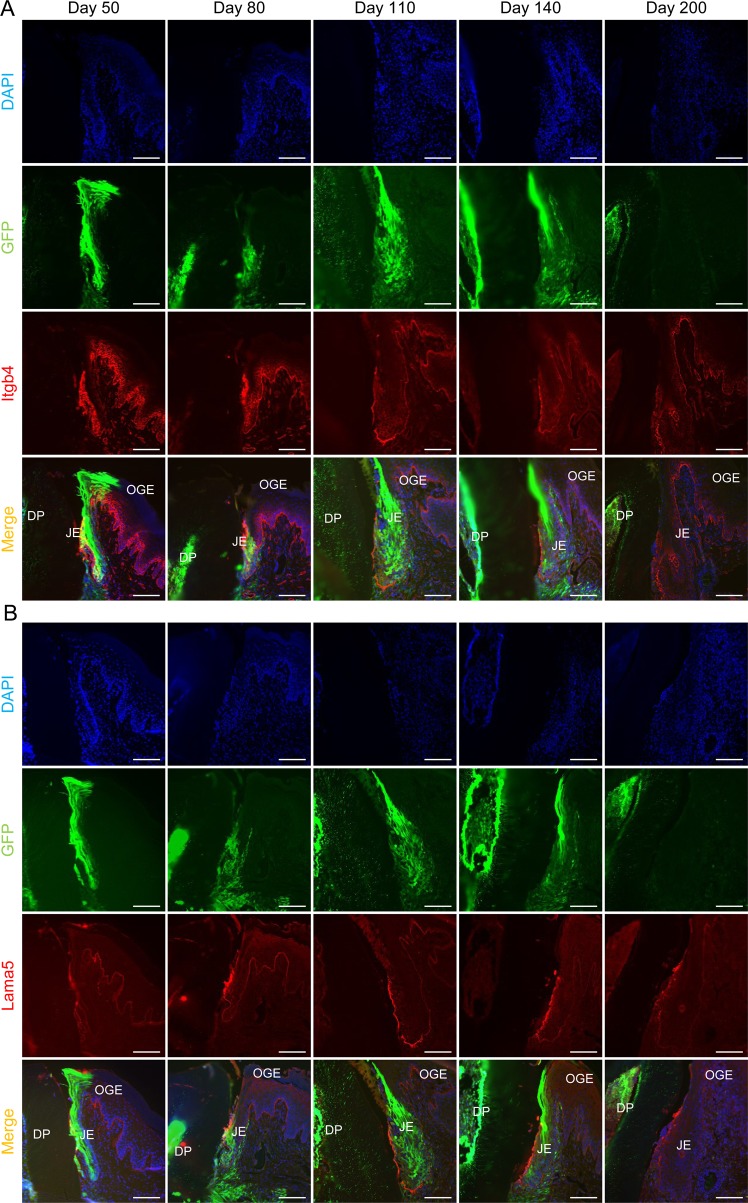
Itgb4 and Lama5 expression in JE replaced with OGE cells. (**A**) Immunofluorescence staining for Itgb4 in transverse sections of JE on day 50, 80, 110, 140, and 200. There was no difference in the expression of Itgb4. (**B**) Immunofluorescence staining for Lama5 in transverse sections of JE on day 50, 80, 110, 140, and 200. There was no difference in the expression of Lama5. Representative images were taken from three independent mouse samples (*n* = 3). Scale bars represent 100 μm. Abbreviations: OGE, oral gingival epithelium; JE, junctional epithelium; DP, dental pulp.

Normally, the JE attaches to the enamel surface with hemidesmosomes mainly consisting of laminin 5 (Lama5) and integrin α6β4. To examine whether adhesion is changed after or before the replacement of JE derived from the odontogenic epithelium with OGE, we detected the expression of integrin β4 (Itgb4) and Lama5 in the JE at each time point. The expression of Itgb4 and Lama5 was detected in the EBL and IBL both before and after the replacement (Fig. 2A,B) and was determined to be independent of JE origins, indicating that the attachment of the JE to the enamel surface might be crucially involved in the expression of both Itgb4 and Lama5. In addition, the microscopic structure of the replaced JE appeared to be similar to that of the JE derived from odontogenic cells (Fig. 2A,B). However, it still remains unclear whether there are significant functional differences between odontogenic and replaced JE. Therefore, our plan is to explore this in further investigations in the future.

**Figure 3:**
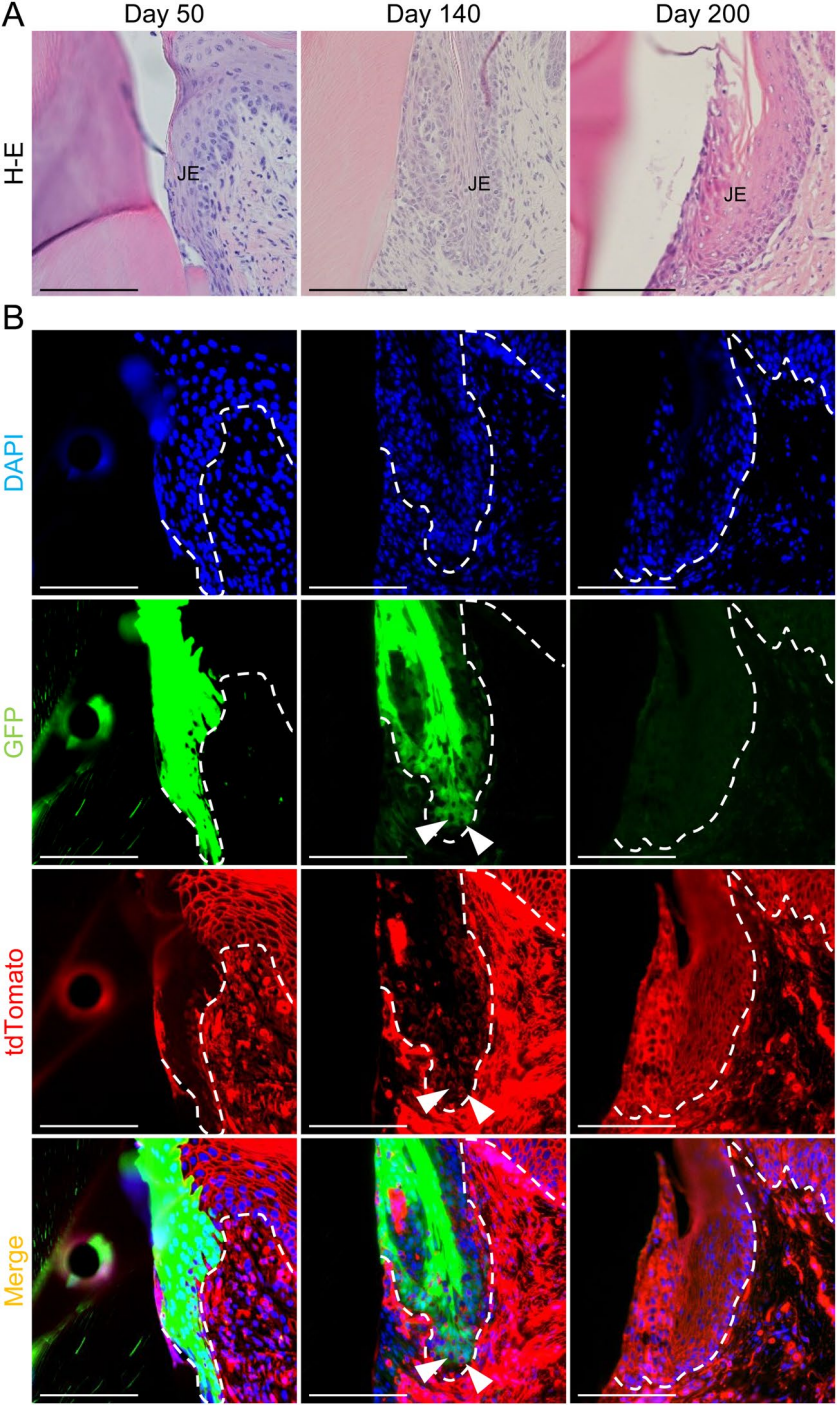
JE derived from odontogenic epithelium was replaced by OGE cells. (**A**,**B**) H&E stained (upper row) and fluorescence images (lower row) of transverse sections of periodontal tissue around the erupted GFP-positive teeth in ROSA^mT/mG^ mice on day 50, 140, and 200 after transplantation. (**A**) No obvious morphological changes in JE were found during the 200-day observation. (**B**) JE consisted entirely of GFP-positive cells on day 50. On day 140, tdTomato-positive cells were observed in the basal cell layer adjacent EBL (white arrowheads). On day 200, almost all of the JE consisted of tdTomato-positive recipient cells. GFP-positive cells were not detected in the JE. Dotted lines show the basement membrane. Scale bars represent 200 μm.

These data suggest that the JE derived from odontogenic cells was replaced by OGE. However, we could not completely rule out the possibility that GFP fluorescence in GFP-positive JE cells was reduced for a long time after the transplantation. Therefore, to determine whether GFP-negative cells are derived from recipient cells, GFP-positive tooth germs were transplanted into the alveolar bone in C57BL/6-Tg (ROSA^mT/mG^) mice, which ubiquitously express tdTomato red fluorescent. JE consisted entirely of GFP-positive cells on day 50 (Fig. 3A,B). On day 140, tdTomato-positive recipient cells were detected in the basal cell layer adjacent to the external basal lamina. On day 200, the JE had been completely replaced by tdTomato-positive recipient cells. These results were almost the same as those obtained using WT mice as recipients. Therefore, we concluded that the JE derived from odontogenic cells was apparently replaced by OGE over time.

### Regenerated JE cells after gingivectomy originate from OGE cells

**Figure 4:**
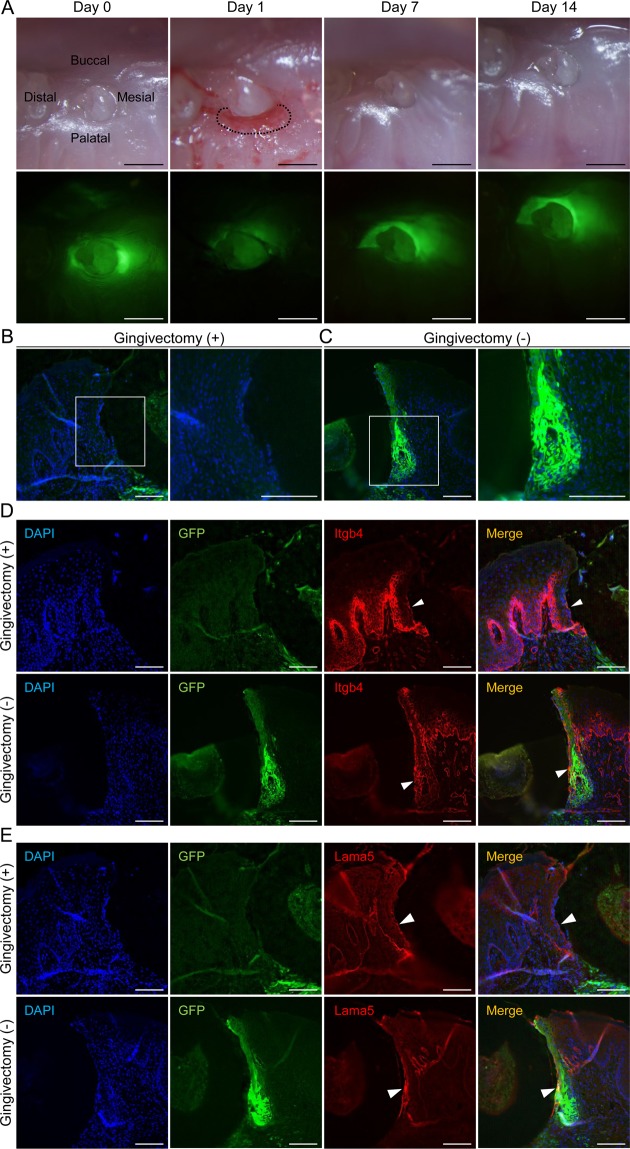
Regenerated JE after gingivectomy originates from OGE cells. (**A**) Representative bright- (upper row) and dark-field (lower row) imaging of the periodontal tissue around erupted GFP-positive teeth in WT mice on day 0, 1, 7, and 14 after palatal gingivectomy. On day 7 and 14, GFP fluorescence was not detected in regenerated JE at the gingivectomy site. GFP fluorescence was found only at the non-gingivectomy site. Scale bars represent 500 μm. (**B**,**C**) Fluorescence images of the JE at the gingivectomy (**B**) and non-gingivectomy (**C**) sites on day 14. Boxed areas are enlarged and shown in right panels. The regenerated JE consisted of GFP-negative cells. Scale bars represent 100 μm. (**D**,**E**) Immunofluorescence staining for Itgb4 (**D**) and Lama5 (**E**) of transverse sections of JE at the gingivectomy and non-gingivectomy sites on day 14. Neither IBLs of the JE showed a difference in Itgb4 and Lama5 expression (white arrowheads). Representative images were taken from three independent mouse samples (*n* = 3). Scale bars represent 100 μm.

Currently, it is believed that the JE regenerates itself when it suffers a minor injury, while it is regenerated by OGE when largely removed by gingivectomy^28^. However, it has not been possible to completely exclude the possibility that remnants of the JE contribute to regeneration after gingivectomy. The tooth germ transplantation technique is a useful method to eliminate this possibility because if remnants of the JE proliferate and regenerate, the regenerated JE should be GFP-positive. Therefore, using this technique, it was determined whether JE derived from the odontogenic epithelium has the ability to regenerate the JE after partial gingivectomy. One day after gingivectomy, no GFP fluorescence was observed at the gingivectomy site (dotted line in Fig. 4A). Seven days after gingivectomy, the surgical site was covered with epithelium just like before the gingivectomy. Green fluorescence was not detected in the gingiva at the surgical site on days 7 and 14, although fluorescence was detected in the gingiva at the opposite side, where gingivectomy had not been performed (Fig. 4A). Histological analyses were performed 14 days after gingivectomy. GFP-positive cells were detected in the control JE, but not in the regenerated JE (Fig. 4B,C). In addition, to examine whether normal adhesion of the regenerated JE recovered, we examined the expression of Itgb4 and Lama5 in the regenerated JE and the control JE. In the regenerated JE, Itgb4 was detected in the IBL and EBL and close to the basal JE cells (white arrowheads in Fig. 4D). Lama5 was also detected in the IBL and EBL of the regenerated JE (white arrowheads in Fig. 4E). Therefore, we were able to verify the normal adhesion of the regenerated JE to the enamel surface through adhesion molecules consisting of hemidesmosomes (Fig. 4D,E). These results suggest that the regenerated JE after partial gingivectomy arose not from residual JE cells but rather from OGE.

### Characterization of odontogenic epithelium-derived JE and OGE-derived JE

**Figure 5:**
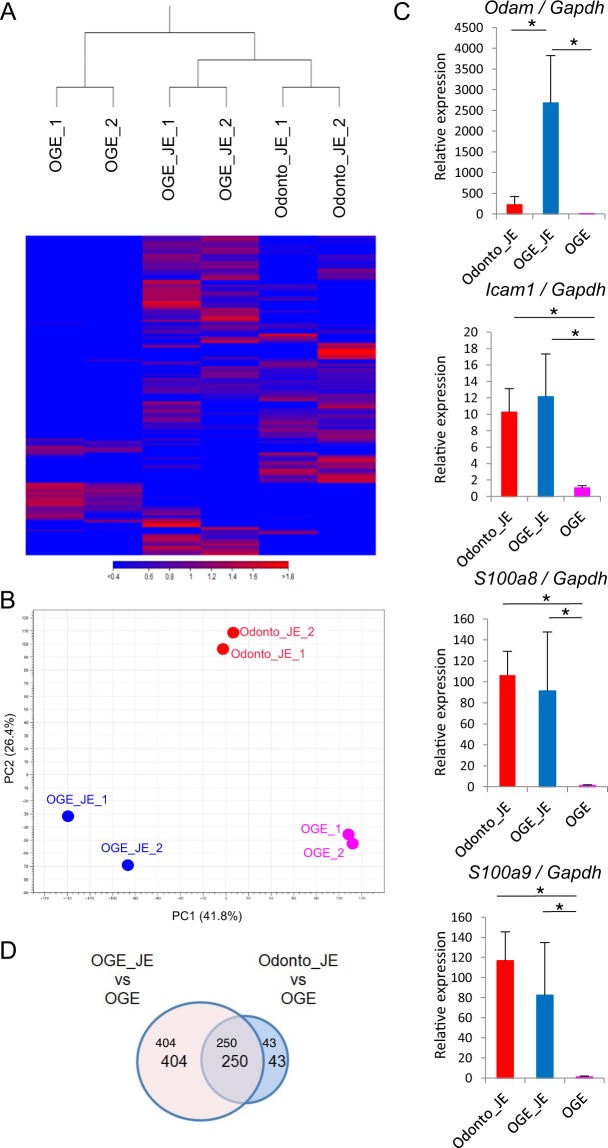
Gene expression of odontogenic epithelium–derived JE and OGE-derived JE. (**A**) Hierarchical cluster analysis based on global gene expression examined by RNA sequencing. Data shown are for odontogenic epithelium–derived JE, OGE-derived JE, and palatal OGE. (**B**) Principle component analysis based on global gene expression examined by RNA sequencing (*n* = 2 in each group). (**C**) Real-time RT-PCR verification of the RNA sequencing data. Gene expression levels of JE-specific genes in Odonto_JE, OGE_JE, and palatal OGE were normalized to *Gapdh*. The data is shown as mean ± s.d. of 3 independent experiments (mean + s.d. of *n* = 3 mice per group). (**D**) Venn diagram illustrating the overlap between the up-regulated genes Odonto_JE compared with OGE and OGE_JE compared with OGE in RNA-seq analysis (FPKM ≥5; fold change ≥5). Abbreviations: Odonto_JE, odontogenic epithelium-derived junctional epithelium; OGE_JE, Oral gingival epithelium-derived junctional epithelium.

Based on analysis of tissue after partial gingivectomy, regenerated JE was found to be derived from OGE rather than odontogenic epithelium. To precisely characterize the odontogenic epithelium–derived JE and OGE-derived JE, we compared the gene expression profiles between odontogenic epithelium–derived JE dissected from mice with GFP-positive tooth germ transplantation, OGE-derived JE dissected from mice 30 days after gingivectomy, and OGE from the palatal region via RNA sequencing (Table S1 and Data S1). Through hierarchical clustering analysis, the odontogenic epithelium-derived JE and the OGE-derived JE were grouped into the same cluster, but the palatal OGE was not (Fig. 5A). On the other hand, based on principal component analysis (PCA), the gene expression patterns of the odontogenic epithelium–derived JE, OGE-derived JE, and palatal OGE were dissimilar (Fig. 5B). Interestingly, several JE-specific genes such as odontogenic genes, ameloblast-associated (*Odam*), intercellular adhesion molecule 1 (*Icam1*), S100 calcium-binding protein A8 (*S100a8*), and S100 calcium-binding protein A9 (*S100a9*) were commonly upregulated in odontogenic epithelium-derived JE and OGE-derived JE relative to OGE (Fig. 5C). Especially *Odam*, the expression in OGE-derived JE cells was higher than odontogenic epithelium-derived JE cells. Next, to analyze the difference between odontogenic epithelium-derived JE and OGE-derived JE, we conducted comprehensive analysis of profiling data using the Venn diagrams and Gene Ontology (GO) enrichment analysis. The Venn diagram revealed that 250 genes are commonly upregulated in OGE- and odontogenic epithelium-derived JE cells compared to OGE. In contrast, 404 genes were expressed in OGE-derived JE cells but not in odontogenic epithelium-derived JE cells, while 43 genes were specific only to odontogenic epithelium-derived JE cells (Fig. 5D). GO enrichment analysis demonstrated that some OGE-derived JE cell-specific genes are associated with inflammatory response and chemotaxis (Data S2).

## Discussion

In the present study, we directly revealed that JE derived from the odontogenic epithelium was replaced by OGE cells over time. In addition, JE that regenerated after gingivectomy originated from OGE cells. OGE-derived JE expressed JE-specific genes, but did not have the same gene expression profile as odontogenic epithelium–derived JE.

Periodontal disease is caused by bacteria found in dental plaque and many other factors, including local and systemic immunoinflammatory responses and environmental factors^8,11^. JE attaches to the tooth surface and forms a defensive line against periodontal bacterial infection. The JE is a non-keratinized epithelium and has wide intercellular spaces that are easily infiltrated by inflammatory cells such as neutrophils and monocytes. Thus, the specific features of the JE are expected to be important barriers against bacterial infections. Therefore, functional changes to the JE may affect the progression of periodontal diseases. Importantly, based on previous histological analyses, the primary JE is believed to be formed by the fusion of the reduced enamel epithelium with the oral epithelium and is gradually replaced by the oral epithelium, suggesting that the functions of the JE might change over the course of a lifetime. To date, studies have shown only indirect evidence for this because there have been no tracing methods to identify the origins of the JE. In the present study, we directly determined the origin of the JE using a tooth germ transplantation technique. JE turnover is reported to be 4–6 days based on the mitotic index and radiography in marmosets and monkeys^13,29^. Furthermore, BrdU-positive cells have been detected in the basal cells of the JE after 2 h and are more distinct after 48 h^30^. This means that the JE has the potential to self-renew. Consistent with this, our previous report also demonstrates the same proliferative potential of the JE, suggesting that the JE generated by our technique is an appropriate model for evaluating the characteristics of normal JE.

In the present results, the JE derived from odontogenic cells was indeed replaced gradually by OGE cells from the basal layer over the course of the 140 days after transplantation of GFP-positive tooth germs. Consequently, JE derived from odontogenic epithelium was mostly replaced by OGE cells after 200 days. Consistent with this, when GFP-positive tooth germs were transplanted into the alveolar bone in ROSA^mT/mG^ mice, similar results were obtained. Therefore, we concluded that odontogenic epithelium–derived JE maintained the potential for self-renewal for some time before finally losing it in part or entirely. On the contrary, in the lingual epithelium, multicolor lineage tracing has demonstrated that one stem cell per interpapillary pit survives long-term^31^. The replacement of odontogenic epithelium–derived JE with OGE may be driven by several mechanisms, such as loss of the self-renewal potential of the odontogenic epithelium–derived JE due to aging or cell competition between odontogenic epithelium–derived JE cells and OGE cells, although the exact mechanisms remain unclear. Therefore, further investigation is necessary in the near future.

There are many reports of restoration after gingival resection^32–36^. In addition, Masaoka *et al*. reported no obvious morphologic changes in the JE between sites subjected to gingivectomy and control sites and the expression of Itgb4 and Lama5 in regenerated gingiva 14 days after mouse gingival resection^37^. These reports do not show the origin of repaired cells. Our study of gingival resection of tooth germ transplantation directly determined that the origin of regenerated JE was OGE cells. Therefore, we are able to confirm that remnants of odontogenic epithelium-derived JE did not contribute to regeneration of the JE after gingivectomy, which was previously unclear. Furthermore, regenerated JE began to express Itgb4 and Lama5. Regenerated JE showed a similar structure to the odontogenic epithelium–derived JE. Additionally, the regenerated JE was considered to have acquired a normal structure. Several studies have reported that cells directly attached to the teeth (DAT cells) are involved in the adhesion of the enamel surface layer^38–40^. In electron microscopy analyses, DAT cells have been found to have numerous microvilli-like structures on the cell surface, which firmly adhere to the tooth surface and migrate DAT cells from the apical to the coronal side. In addition, Itgb4 expression is involved in DAT cell adhesion and migration as well as epithelial turnover^38^. In the present study, migration of DAT cells was not detected at the resected site, and it was revealed that recovery of odontogenic epithelium-derived JE did not occur after gingivectomy.

To characterize JE derived from different origins, we compared the gene expression profiles of the odontogenic epithelium-derived JE and OGE-derived JE, which were resected from transplanted teeth and regenerated JE, via RNA sequencing. Surprisingly, several genes, such as *Odam*, *Icam1*, *S100a8*, and *S100a9*, were commonly upregulated in odontogenic epithelium–derived JE and OGE-derived JE relative to OGE. Importantly, *Odam*, an ameloblast-associated gene, was found to be a common upregulated gene in OGE- and odontogenic epithelium-derived JE cells. *Odam* is also reported to be specific to JE cells^33^. Therefore, the OGE might have the potential to partly acquire the characteristics of odontogenic epithelium-derived JE. However, it was unexpected that the *Odam* expression in OGE-derived JE cells was much higher than that in odontogenic epithelium-derived JE cells. The Odam-ARHGEF5-RhoA signaling pathway is reported to play a significant role in tooth–cell adhesion. Moreover, laminin, which activates integrin-mediated *Odam* signaling, participates in proliferation, differentiation, and actin rearrangement during JE formation^41–45^. We speculate that the high *Odam* expression in the replaced JE might be due to proliferation, differentiation, and actin rearrangement during OGE-derived JE formation after gingivectomy. Consistent with this, GO enrichment analysis demonstrated that some OGE-derived JE cell-specific genes are associated with inflammatory response and chemotaxis. Therefore, these data suggested the possibility that OGE-derived JE cells were still in the process of wound healing, although 30 days after gingivectomy, OGE-derived JE cells appear morphologically normal. PCA revealed that the gene expression patterns of odontogenic epithelium-derived JE and OGE-derived JE were not similar. Therefore, it is an important thing to clarify whether gene expression patterns of OGE-derived JE gradually become similar to those of odontogenic epithelium-derived JE or not. This will be examined in future studies.

## Materials and Methods

### Animals

C57BL/6 N and C57BL/6N-Tg (CAG-EGFP) mice^46^ were purchased from Sankyo Labo service, Inc. (Tokyo, Japan). C57BL/6-KI (ROSA^mT/mG^) mice were purchased from The Jackson Laboratory^47^. All mice were born alive and maintained under specific pathogen-free (SPF) conditions. The mouse experiments were approved by and conducted according to the guidelines of the Showa University Animal Care and Use Committee (number 18007).

### GFP-positive tooth germ transplantation method

The tooth germ transplantation method was performed as previously described^48^. Briefly, molar tooth germs were collected from the mandibles of C57BL/6-Tg (CAG-EGFP) mice on E14. The collected molar tooth germs were placed in a gel drop of Cellmatrix type I-A (Nitta Gelatin). Then, the tooth germs were cultured in Dulbecco’s modified Eagle’s medium (DMEM) (Wako) Supplemented with 10% fetal bovine serum (FBS) and 1% penicillin-streptomycin at 37 °C in a humidified atmosphere at 5% CO_2_ for 4 d. The upper first molars of 3-week-old C57BL/6 N (WT) and C57BL/6-Tg (ROSA^mT/mG^) mice were extracted under deep anesthesia and then the alveolar bones at the tooth extraction sites were allowed to heal for 3 weeks. A hole approximately 1.0 mm in diameter was made in the exposed alveolar bone surface of C57BL/6 N and ROSA^mT/mG^ mice and the tooth germ was transplanted into the hole.

### Histological analyses after gingivectomy

The palatal gingiva of the transplanted teeth of WT mice were excised using a surgical knife (No. 11; Feather, Osaka, Japan) under deep anesthesia. The buccal side served as a control. A piece of gingival tissue including GFP-positive JE was resected from the medial to the distal side of the transplanted tooth according to a method described in a previous study^37^. The gingival tissue around the transplanted teeth was observed using a fluorescence stereomicroscope on days 0, 1, 7, and 14 after gingivectomy (SZX7; Olympus, Tokyo, Japan). Histological analyses of the JE around the transplanted teeth in WT mice were performed 14 d after gingivectomy.

### Micro-CT scanning

Scanning was performed using a micro-CT device in an *in vivo* 3D µCT system according to the manufacturer’s protocol (R_mCT2, Rigaku Co., Ltd., Tokyo, Japan).

### Immunohistological analyses

The maxillae were dissected and fixed with 4% paraformaldehyde for 6 h at 4 °C after decalcification with 10% ethylenediaminetetraacetic acid (EDTA) for 2 weeks at 4 °C. The specimens were embedded in optimal cutting temperature compound (Sakura) and then immediately snap-frozen in liquid nitrogen-cooled isopentane. The frozen sections were cut using a cryomicrotome (Microm) to 5 μm thickness in the buccal–lingual direction. Hematoxylin and eosin (H&E) or immunofluorescence staining were performed on the sections. For immunofluorescence staining, the frozen sections were air-dried for 10 min, washed with tris-buffered saline (TBS), and pre-incubated with blocking solution (Dako) for 10 min. The sections were incubated with an anti-integrin β4 rat polyclonal antibody (Cat. No. ab25254; 1:100: Abcam) for 1 h and an anti-laminin 5 rabbit monoclonal antibody (Cat. No. ab14509; 1:200: Abcam) for 2 h at room temperature. After washing in TBS, the sections were incubated for 30 min at room temperature with an anti-rabbit IgG antibody conjugated with Alexa 594 or an anti-rat IgG Alexa 594 of donkey origin (1:200 dilution; Molecular Probes). After counterstaining with 49, 6-diamidino-2-phenylindole dihydrochloride (DAPI; 1:500 dilution; Dojindo), all specimens were examined and photographed (BZ-9000 fluorescence microscope, Keyence).

### RNA sequencing

Odontogenic epithelium–derived JE was collected from the GFP-positive gingiva around the transplanted teeth. Odontogenic epithelium-derived JE and OGE-derived JE were collected from GFP-positive tooth-transplanted WT mice using a surgical knife (No. 11; Feather, Osaka, Japan) with a stereomicroscope under deep anesthesia. The odontogenic epithelium-derived JE was resected as a GFP-positive gingiva surrounding the crown of the transplanted tooth. The OGE-derived JE collected the regenerative region around the transplanted tooth 30 days after gingivectomy, while the OGE collected the gingiva distant from the junctional epithelium. Under the stereomicroscope, mesenchymal tissue was surgically removed as much as possible (*n* = 2 mice per group. One specimen for one biological replicate.).

OGE-derived JE was resected from GFP-negative regenerated JE on day 30 after gingivectomy. Non-junctional OGE was collected from palatal gingiva. Total RNA was extracted from tissue using the RNeasy Plus Mini Kit (Qiagen) following the manufacturer’s instructions. A library for RNA sequencing was prepared using the TruSeq ChIP Sample Prep Kit according to the manufacturer’s instructions. Paired-end sequencing (read length: 101 + 101) was carried out using the Illumina HiSeq 2500 system. The sequence reads were aligned to the mouse reference genome (mm10) using Tophat 2.0.13 (bowtie2-2.2.3), which can adequately align reads to the location, including splice sites, in the genome sequence. Sequence data were analyzed using HCS version 2.2.58, RTA version 1.18.64, bcl2fastq-1.8.3, and CLC Genomics Workbench.

### Reverse transcription PCR (RT-PCR)

Total RNA was extracted from tissue samples using the RNeasy Mini Kit Plus (Qiagen) according to the manufacturer’s instructions (*n* = 3 biological replicate). Complementary DNA (cDNA) was generated by reverse transcription using SuperScript VILO Master Mix (Thermo Fisher). The cDNA was mixed with Power SYBR^®^ Green PCR Master Mix (Thermo Fisher) and specific gene primers. PCR was performed using the Gene Amp PCR System 9700 (Thermo Fisher) with the primer sequences listed below. The amplification conditions consisted of an initial denaturation step at 95 °C for 10 min, followed by 40 cycles of denaturation at 95 °C for 15 sec, annealing and elongation at 60 °C for 1 min. The housekeeping gene glyceraldehyde-3-phosphate dehydrogenase (*Gapdh*) was used as an endogenous control.

*Odam*, 5′-TTGACAGCTTTGTAGGCACA-3′ and 5′-GACCTTCTGTTCTGGAGCAA-3′

*Icam1*, 5′-CAATTTCTCATGCCGCACAG-3′ and 5′-AGCTGGAAGATCGAAAGTCCG-3′

*S100a8*, 5′-AAATCACCATGCCCTC-3′ and 5′-CCCACTTTTATCACCA-3′

*S100a9*, 5′-ATACTCTAGGAAGGAA-3′ and 5′-TCCATGATGTCATTTA-3′

*Gapdh*, 5′-TGGCAAAGTGGAGATTGTTGCC-3′ and

5′-AAGATGGTGATGGGCTTCCCG-3′

### Statistical analysis

All data are expressed as the mean ± s.d. Differences between the groups were evaluated by one-way analysis of variance (ANOVA), followed by Tukey’s honestly significant difference (HSD). p < 0.05 was considered significant.

## Supplementary information

Supplementary Information

Dataset 1

Dataset 2
