# Supplementary material for: Visualization of junctional epithelial cell replacement by oral gingival epithelial cells over a life time and after gingivectomy
Source: Sci Rep. 2019 May 21;9:7640. doi: 10.1038/s41598-019-44065-x (PMC6529510; doi:10.1038/s41598-019-44065-x)
Supplement: Supplementary file 1 — Supplementary Information [file 41598_2019_44065_MOESM1_ESM.docx]

**Supplementary Information**

**Visualization of junctional epithelial cell replacement**

**by oral gingival epithelial cells** **over a life time and after gingivectomy**

Mayu Kato^1, 2^, Junichi Tanaka^2^, Ryo Aizawa^1^, Sara Yajima-Himuro^1^, Tatsuaki Seki^1^, Keisuke Tanaka^1, 2^, Atsushi Yamada^3^, Miho Ogawa^4, 5^, Ryutaro Kamijo^3^, Takashi Tsuji^4, 5^, Kenji Mishima^2^, Matsuo Yamamoto^1, *^

^1^ Department of Periodontology, School of Dentistry, Showa University, 2-1-1 Kitasenzoku, Ohta-ku, Tokyo 145-8515, Japan,

^2^ Division of Pathology, Department of Oral Diagnostic Sciences, School of Dentistry, Showa University, 1-5-8 Hatanodai, Shinagawa-ku, Tokyo 142-8555, Japan,

^3^ Department of Biochemistry, School of Dentistry, Showa University, 1-5-8 Hatanodai, Shinagawa-ku, Tokyo 142-8555, Japan,

^4^ Laboratory for Organ Regeneration, RIKEN Center for Biosystems Dynamics Research (BDR), Kobe, Hyogo 650-0047, Japan,

^5^ Organ Technologies Inc., Tokyo 101-0048, Japan.

Correspondence and requests for materials should be addressed to M.Y. (yamamoto-m@dent.showa-u.ac.jp).

Supplementary Table S1: Top 50 genes up-regulated in odontogenic epithelium-derived JE and the OGE-derived JE to OGE.

| Gene_id | Gene_name | OGE (FPKM) | OGE_JE (FPKM) | Odonto_JE (FPKM) | Fold change (OGE_JE/OGE) |
| --- | --- | --- | --- | --- | --- |
| ENSMUSG00000050635 | *Sprr2f* | 0.052727 | 1665.918 | 569.599 | 31595.15997 |
| ENSMUSG00000056054 | *S100a8* | 0.519389 | 14583.92 | 2642.04 | 28078.99282 |
| ENSMUSG00000042212 | *Sprr2d* | 0.2844967 | 7232.59 | 2053.475 | 25422.40385 |
| ENSMUSG00000028186 | *Uox* | 0.0097289 | 191.30935 | 10.18 | 19664.02677 |
| ENSMUSG00000056071 | *S100a9* | 1.1124555 | 20789.71 | 2171.325 | 18688.1273 |
| ENSMUSG00000050092 | *Sprr2b* | 0.03997825 | 470.65 | 38.83004 | 11772.65138 |
| ENSMUSG00000096035 | *Odaph* | 0.08095725 | 944.49 | 552.4025 | 11666.5277 |
| ENSMUSG00000042157 | *Sprr2i* | 0.4741565 | 3470.645 | 1171.7465 | 7319.619155 |
| ENSMUSG00000029417 | *Cxcl9* | 0.0367514 | 260.109 | 60.93075 | 7077.526298 |
| ENSMUSG00000074445 | *Sprr2a3* | 0.13835025 | 847.5685 | 43.591995 | 6126.252031 |
| ENSMUSG00000029371 | *Cxcl5* | 0.12178445 | 670.68 | 171.2382 | 5507.107024 |
| ENSMUSG00000063779 | *Chil4* | 0.08758275 | 316.6705 | 34.1529 | 3615.672036 |
| ENSMUSG00000046259 | *Sprr2h* | 0.4480165 | 1463.703 | 401.474 | 3267.073869 |
| ENSMUSG00000057346 | *Apol9a* | 0.01722195 | 53.7449 | 37.6412 | 3120.72094 |
| ENSMUSG00000009580 | *Odam* | 0.161553 | 314.476 | 581.095 | 1946.580998 |
| ENSMUSG00000045027 | *Prss22* | 0.1063353 | 158.59415 | 28.0535 | 1491.45345 |
| ENSMUSG00000029322 | *Plac8* | 0.302949 | 447.7865 | 17.7457 | 1478.092022 |
| ENSMUSG00000021749 | *Oit1* | 0.0215747 | 28.33705 | 18.21245 | 1313.438889 |
| ENSMUSG00000049723 | *Mmp12* | 0.03956375 | 48.06755 | 48.59215 | 1214.93918 |
| ENSMUSG00000066861 | *Oas1g* | 0.03861785 | 43.30795 | 18.2937 | 1121.449019 |
| ENSMUSG00000058427 | *Cxcl2* | 0.0955925 | 105.55705 | 22.96325 | 1104.239872 |
| ENSMUSG00000027925 | *Sprr2j-ps* | 0.281271 | 305.5105 | 60.45585 | 1086.178454 |
| ENSMUSG00000034855 | *Cxcl10* | 0.256197 | 269.521 | 69.04845 | 1052.006854 |
| ENSMUSG00000027514 | *Zbp1* | 0.0674671 | 61.90565 | 22.433 | 917.5679702 |
| ENSMUSG00000060550 | *H2-Q7* | 0.669547 | 531.858 | 158.971 | 794.3549893 |
| ENSMUSG00000022602 | *Arc* | 0.0249292 | 18.66625 | 7.51614 | 748.7705181 |
| ENSMUSG00000026536 | *Mnda* | 0.0752392 | 49.7453 | 9.33024 | 661.1620007 |
| ENSMUSG00000047104 | *Pbp2* | 0.10921965 | 71.6612 | 12.33755 | 656.1200297 |
| ENSMUSG00000020407 | *Upp1* | 0.5959305 | 372.547 | 47.91025 | 625.1517585 |
| ENSMUSG00000043263 | *Ifi209* | 0.0588391 | 34.5508 | 8.996865 | 587.208166 |
| ENSMUSG00000104713 | *Gbp6* | 0.05905045 | 24.57745 | 17.842455 | 416.2110534 |
| ENSMUSG00000015134 | *Aldh1a3* | 0.5701845 | 236.771 | 122.71475 | 415.2533084 |
| ENSMUSG00000041827 | *Oasl1* | 0.111497 | 42.3106 | 14.2347 | 379.4774747 |
| ENSMUSG00000068246 | *Apol9b* | 0.1237195 | 44.1504 | 17.8965 | 356.8588622 |
| ENSMUSG00000035692 | *Isg15* | 1.218125 | 420.1975 | 271.688 | 344.9543356 |
| ENSMUSG00000079017 | *Ifi27l2a* | 4.964435 | 1656.972 | 738.246 | 333.7684953 |
| ENSMUSG00000030666 | *Calcb* | 0.0889532 | 29.35655 | 58.7756 | 330.0224163 |
| ENSMUSG00000009185 | *Ccl8* | 0.543731 | 179.2135 | 23.9274 | 329.599563 |
| ENSMUSG00000006345 | *Ggt1* | 0.292126 | 93.06865 | 57.08545 | 318.5907793 |
| ENSMUSG00000079451 | *Tmprss11g* | 0.9025525 | 283.724 | 95.8192 | 314.3573366 |
| ENSMUSG00000034459 | *Ifit1* | 0.528938 | 160.155 | 102.30075 | 302.7859598 |
| ENSMUSG00000078920 | *Ifi47* | 0.3565835 | 103.22565 | 31.85185 | 289.4852117 |
| ENSMUSG00000017737 | *Mmp9* | 0.2103675 | 55.83345 | 11.809745 | 265.4091055 |
| ENSMUSG00000063727 | *Tnfrsf11b* | 0.164613 | 40.3259 | 19.08096 | 244.9739692 |
| ENSMUSG00000025491 | *Ifitm1* | 0.4204455 | 100.4015 | 16.465215 | 238.7978941 |
| ENSMUSG00000052776 | *Oas1a* | 0.26033965 | 62.01825 | 42.282 | 238.2205323 |
| ENSMUSG00000073409 | *H2-Q6* | 0.7161805 | 166.02 | 72.8845 | 231.8130695 |
| ENSMUSG00000033355 | *Rtp4* | 0.5854475 | 135.5565 | 94.1005 | 231.543392 |
| ENSMUSG00000032496 | *Ltf* | 0.4262175 | 97.5541 | 117.5322 | 228.8833753 |
| ENSMUSG00000046733 | *Gprc5a* | 0.1117981 | 25.093 | 13.47215 | 224.4492527 |
